# Applications of Engineering Techniques in Microvasculature Design

**DOI:** 10.3389/fcvm.2021.660958

**Published:** 2021-04-26

**Authors:** Aleen Al Halawani, Ziyu Wang, Linyang Liu, Miao Zhang, Anthony S. Weiss

**Affiliations:** ^1^School of Life and Environmental Sciences, University of Sydney, Sydney, NSW, Australia; ^2^Charles Perkins Centre, University of Sydney, Sydney, NSW, Australia; ^3^School of Biomedical Engineering, University of Sydney, Sydney, NSW, Australia; ^4^Sydney Nano Institute, University of Sydney, Sydney, NSW, Australia

**Keywords:** microvasculature, extracellular matrix, bioprinting, micropatterning, porous scaffolds, vascularized organoids, elastin, tropoelastin

## Abstract

Achieving successful microcirculation in tissue engineered constructs *in vitro* and *in vivo* remains a challenge. Engineered tissue must be vascularized *in vitro* for successful inosculation post-implantation to allow instantaneous perfusion. To achieve this, most engineering techniques rely on engineering channels or pores for guiding angiogenesis and capillary tube formation. However, the chosen materials should also exhibit properties resembling the native extracellular matrix (ECM) in providing mechanical and molecular cues for endothelial cells. This review addresses techniques that can be used in conjunction with matrix-mimicking materials to further advance microvasculature design. These include electrospinning, micropatterning and bioprinting. Other techniques implemented for vascularizing organoids are also considered for their potential to expand on these approaches.

## Introduction

There are key features that are integral to the engineered matrix and are required for fabricating 3D vascularized tissues. These include mechanical performance, geometry, biodegradability, cell-matrix interactions and subsequent remodeling capacity (1). Cells that interact with the construct must be able to attach, proliferate, spread, and establish chemoattractant gradients to facilitate infiltration by further cells (2). Furthermore, the porosity of the native extracellular matrix (ECM) should be recapitulated in the engineered matrix (3). These properties collectively provide an environment that simulates the native ECM to enhance spatiotemporal and biological cell behavior.

The native ECM environment can be divided into basement membrane and interstitial matrix. The basement membrane acts as a barrier to separate cells from surrounding connective tissue, provides structural support, and modulates cell function. It is primarily composed of glycoproteins and fibrous proteins such as collagen IV and laminin (4, 5). The interstitial matrix consists of proteoglycans, which can sequester water, and fibrous proteins such as collagen and fibronectin that provide broader structural scaffolding (6). Laminin I, a basement membrane protein, is largely responsible for endothelial cell (EC) quiescence whereas contact with collagen I in the interstitial matrix drives capillary tube morphogenesis. Redeposition of basement membrane proteins by mural and ECs, after the network is formed, marks network stability and maturation (7). Furthermore, matrix density and rigidity heavily influence capillary network development by enabling cells to generate adequate traction forces (8). Such contractile forces translate changes in matrix mechanics to changes in cell behavior. In addition to mechanical matrix characteristics that influence vascular network formation, ECM degradation during wound healing allows generation of bioactive peptides and release of trapped growth factors that promote angiogenesis at the site of injury. Collectively, these matrix properties are crucial in guiding vascularization and maintaining vascular networks so should be reflected in engineered matrix materials for developing vascularized tissue.

Multiple engineering approaches such as fabricating porous scaffolds, micropatterning, and 3D bioprinting have been developed to facilitate the process of neovascularisation and angiogenesis by fabricating pores or microchannels. Furthermore, natural polymers such as elastin, collagen, and fibronectin have been used either as a sole component or in combination with synthetic polymers to fabricate functionalized biomaterials for use in combination with these types of approaches. In addition to providing structural support, these materials also provide cell-adhesive ligands and cues that are inherently abundant in the ECM to enhance the biological function of the engineered matrices. It is well-appreciated that matrix components dictate microvasculature formation and architecture. This has been reviewed elsewhere (3, 9). Recently, advances in matrix engineering have allowed the development of vascularized organoids, which can be further used in association with bioinks for additive manufacturing of vascularized engineered tissue. In this review, we describe the various engineering techniques that partly implement natural polymers for the fabrication of vascularized matrices and highlight the potential of using elastin-derived materials for such purposes.

## Porous Scaffolds and Vascularization

Although the polymers used for fabricating porous scaffolds can exhibit a certain degree of porosity, relying on this alone is not sufficient. The scaffold must exhibit some durability and strength to withstand fabrication and implantation, where substantial porosity can weaken the construct while allowing for tissue invasion and vascularization. This means that denser regions should be punctuated by defined porosity, in order to retain construct durability and strength. To further incorporate pores into scaffolds, engineering techniques such as gas foaming, porogen leaching, and electrospinning are implemented.

Pore size in an engineered porous scaffold refers to pore geometry achieved by implementing various techniques to incorporate mesoscopic (>50 μm) and microscopic (1–50 μm) pores within the scaffold for facilitating tissue invasion (10). Achieving a hierarchical distribution of pores throughout a scaffold is crucial as pore size influences tissue shape, cell function and the rate and depth of tissue invasion. Engineered pores must exhibit a desirable size for vascularization. Large pore sizes (>200 μm) allow for deep vascularization and tissue invasion, but the formed blood vessels are larger than those observed at high density with a small pore size (>200 μm) (11). However, a small pore size yields poor tissue invasion. Pore size alone is insufficient to enhance the biological potential of the scaffold. Pore interconnectivity links adjacent pores, allowing for a continuous flow and connection between forming capillary tube segments. A porous scaffold with poor interconnectivity does not support cell attachment and migration (12, 13). The size and number of blood vessels formed within a macroporous scaffold increases when the interconnection size between pores increases to 320 μm (12).

There are multiple conventional techniques that are used to incorporate pores into a scaffold. These include dense gas foaming, where the pore size is controlled by adjusting temperature and pressure; and particle leaching, in which the size and shape of the pore is determined by the type and size of porogen particles. Each of these techniques is capable of forming interconnections between pores. However, the high processing temperatures in the porogen leaching process limits the choice of bioactive molecules which need to survive heating. Furthermore, the persistence of residual porogen can limit its translational utility. To address these limitations, advances in electrospinning have allowed for increased control over pore morphology and size.

Electrospinning provides an opportunity to fabricate nanofibers and microfibers from both synthetic and natural polymers, which mimic the native fibrous elements in the ECM. A broad range of synthetic and natural polymers have been utilized for the fabrication of fibrous scaffolds that promote angiogenesis using electrospinning. However, conventional electrospinning techniques have limited potential for cell infiltration in part due to diverse and inconsistent pore sizes (14). This has been addressed in part via core-shell electrospinning which allows angiogenic factors such as vascular endothelial growth factor (VEGF) (15) and connective tissue growth factor (CTGF) (16) to be incorporated into the nanofiber core. Deposited fibers gradually release the housed angiogenic factors upon degradation. Furthermore, melt electrospinning provides for further control over porosity through the use of molten polymers such as polycaprolactone instead of a polymer solution in an organic solvent. This overcomes potential volatility and toxicity issues seen with organic solvents. The polymer melt cools and thus solidifies prior to or on the collector, offering control over fiber placement. The resulting spatial configuration offers improvements in uniformity of pore size and interconnectivity. This results in enhanced angiogenesis, cell infiltration, and tissue repair (17).

## Micropatterned Microvasculature

Micropatterning for vascularization involves the subtraction of predefined patterns within the engineered matrix to create microchannels that resemble a vascular network. It can be classified into two modalities: micromolding (18, 19) and laser degradation (20). Fine-tuned micropatterns on the scaffold provides spatial cues allowing for EC attachment and subsequent 3D-tube formation.

In micromolding, a predesigned microscale mold is first incorporated into a soft material. After the material sets, the mold is removed, leaving hollow microchannels for endothelialization. For example, Teflon coated wires with a diameter of 500 μm incorporated into a silk-based scaffold act as a mold for fabricating microchannels within a thick silk scaffold (7 mm). These channels allowed vascularization within 2 weeks post-implantation in mice (19). However, a simple tubular mold cannot capture the complexity of natural microvasculature within the tissue. More complex molds can be fabricated by soft lithography, which uses visible or UV light to transfer micro-scaled patterns from a photo mask to the surface of a material and can potentially provide shape and size control of the microchannels.

**Figure 1:**
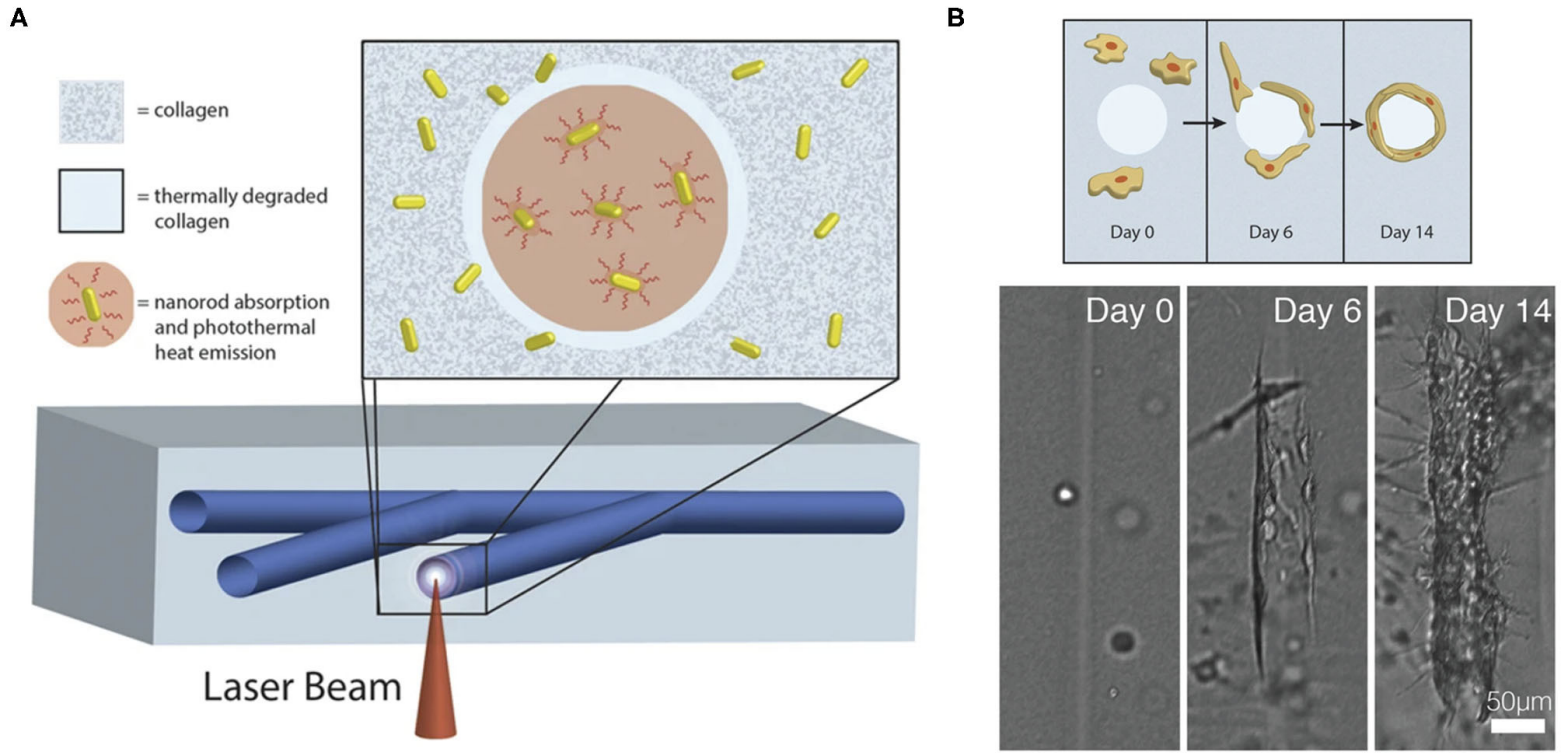
**(A)** Fabrication of computer-generated branching microchannels within a collagen hydrogel using a near-infrared (NIR) laser through photothermal degradation of the collagen. **(B)** Visualization of endothelial cell migration and tube formation in a laser degraded microchannel by brightfield microscopy. Adapted with permission from Hribar et al. (21).

Laser degradation uses energy emitted by a laser beam to degrade hydrogel on the laser focal point path, which moves following a predesigned digital 3D pattern. The degraded hydrogel is then flushed out of the construct to generate microchannels. Hydrogels used for laser degradation can be made from natural polymers such as silk (20), collagen (21, 22) and elastin (22), synthetic polymers such as poly(ethylene glycol)diacrylate (PEGDA) (23), PEG-tetrabicyclononyne (24) or composite materials such as polyethylene glycol (PEG)-fibrinogen (25). Incorporating natural polymers is crucial in providing cell-adhesion ligands for cell-matrix interactions and biochemical signaling in the microenvironment to induce angiogenesis post-implantation. Laser degradation provides a simple way to fabricate complex and high-resolution endothelialized vascular networks with spatiotemporal control (Figure 1) (20, 21, 26). 3D pattern of native microstructure can be obtained through computer-aided reconstruction from ultrasound, CT, and MRI data, and the fabricated highly mimetic microvasculature can potentially minimize remodeling after implantation (26).

Micropatterning provides a path for researchers to optimize the architecture of engineered matrix by exploring cell behavior in a bespoke environment that aims to mimic the native ECM microenvironment (27). For vascularization, perfusable interconnected microchannels can be optimally achieved by laser degradation with more precise spatiotemporal control. In addition to offering flexibility over scaffold design, micropatterning enables modification of the scaffold into defined microdomains with different surface modifications, which allows spatiotemporal control over the healing response through the provided gradient of growth factors. The hollow channels generated by these approaches can serve as pre-vascular molds. During angiogenesis, ECs must burrow throughout the ECM to create a path for forming capillary tubes. These engineering techniques assist in angiogenic events and future vascularization by providing a template for ECs to form capillary tubes.

## Bioprinted Microvasculature

Blood vessels in tissue are three-dimensional (3D) and hierarchical with diameters ranging from 10 μm to 1 cm. This hierarchal organization is crucial for satisfying cellular demands in diverse locations. Traditional approaches such as angiogenesis in 3D porous scaffolds and micropatterning often fail to capture the scaled nature of complex vascular systems in local tissues. For example, angiogenesis in porous scaffolds only allows for the formation of non-hierarchical capillary networks, while micropatterning often results in a 2D system with embedded planar microchannels. Bioprinting is a popular method that allows for the automatic fabrication of 3D hierarchical microvasculature-like microchannels at levels of precision limited by the bioprinter resolution. Various bioprinting techniques such as extrusion bioprinting, inkjet bioprinting, laser-assisted bioprinting, and stereolithography (SLR) have been developed (28), but the fabrication of vascularized tissue constructs are governed by one key concept that employs these techniques - the formation of hollow microchannels within cell-embedded hydrogel constructs to allow for efficient perfusion, which can be typically achieved through sacrificial templates or direct microchannel formation.

**Figure 2:**
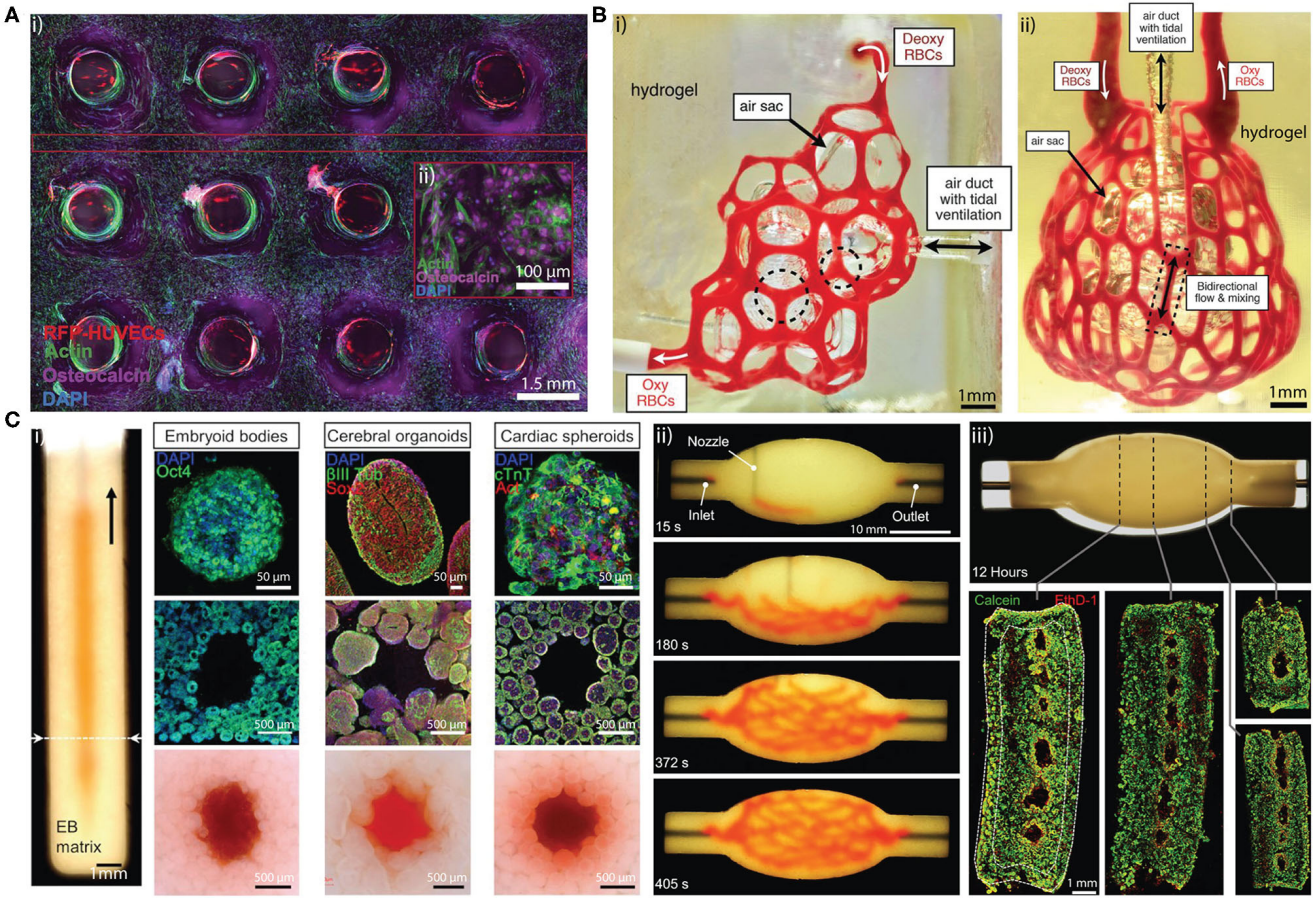
**(A)** (i) Cross sectional confocal microscopy image of a 1 cm-thick osteogenic tissue construct with perfused lattice microchannel after 30 days. (ii) High-resolution image showing osteocalcin (purple) localized within human mesenchymal stem cells, which present symmetric osteoblast-like morphologies. Adapted with permission from Kolesky et al. (31). **(B)** (i) Hierarchical microchannel printed in hydrogel is perfused with red blood cells while ventilating the air sac with oxygen. (ii) Printed hierarchical microchannel mimicking the distal lung subunit. The microchannel is perfused red blood cells while ventilating the air sac with oxygen. Adapted with permission from Grigoryan et al. (38). **(C)** (i) Photograph of a vertical microchannel within an embryoid bodies (EB) matrix. The EB matrix can be substitute by cerebral organoids and cardiac spheroids (top row). The microchannel at the dashed line is visualized by immunostained slices in the (middle row) and bright-field images (bottom row), respectively. (ii) A sequence of images illustrating the printing process of a hierarchical microchannel in EB-based tissue matrix before sacrifice. (iii) Sacrificed hierarchical microchannel after 12 h of perfusion (top) and fluorescent image of LIVE/DEAD (green/red) cells at various cross sections (bottom). Adapted with permission from Skylar-Scott et al. (39).

In a sacrificial approach, a template can be printed with pre-designed 3D structure through extrusion bioprinting (29–31) and selective laser sintering (32). The template is then submerged in a cell-embedded supporting hydrogel (31), or the template can be directly extruded into the supporting hydrogel (29, 33, 34). Subsequently, the template is sacrificed by exploiting its thermal or chemical properties and flushed out of the hydrogel system. For example, Pluronic F127 (30) and gelatin (35) liquidize at 0 and 37°C, respectively, while carbohydrate (32, 36) and salts dissolve in cell culture medium rapidly after immersion. Hollow microchannels are then endothelialized *in vitro* to allow faster inosculation after implantation (31, 37). 3D lattice microchannel networks with endothelial lining (31) (Figure 2A) are most frequently used due to their ability to perfuse the entire cell-embedded hydrogel. These microchannels can serve to guide endothelialization, but this does not always ensure that they will function as a robust microvasculature. While bioprinted hierarchical microchannels can be structurally similar to the native tissue microvasculature (Figure 2B) (38), the surrounding cell-embedded hydrogel is also crucial for the development of tissue-specific vascularized constructs. Tissue-specific cell spheroids or organoids such as cardiac spheroids, embryoid bodies, cerebral organoids have been mixed with ECM mimetic gel such as collagen I and Matrigel to generate granular tissue matrix, which permits the extrusion of sacrificial hierarchical template within the granular tissue matrix (Figure 2C) (39), which demonstrates the potential for rapid generation of patient-specific functional tissue.

Direct formation of microchannels can be achieved through coaxial extrusion, inkjet, light-assisted bioprinting, and SLR. In coaxial extrusion bioprinting, a sacrificial substance such as alginate in the shell flow is crosslinked by the calcium ions diffused from the core flow, forming gelled alginate microchannels (40). Inkjet and light-assisted bioprinting generate bioink droplets, which are deposited into layered ring shapes that are supported by the surrounding hydrogel (33, 41). The droplets can self-assemble to form an almost complete wall for the microchannel. In the SLR approach, the cell-embedded photosensitive hydrogel is crosslinked subsequent to laser light exposure using a predesigned pattern in a layer-by-layer format (42). The uncrosslinked hydrogel is then flushed out, leaving microchannels through the constructs.

## Guiding Organoid Vascularization

Organoids are self-assembled 3D structures derived from embryonic, induced, or adult stem cells that resemble larger organs (43). Since organoids have the same function as their larger counterparts, they can be considered as building blocks toward constructing larger tissues and organs. However, a central challenge in organoid development is vascularization, as diffusion alone is not sufficient to ensure the viability of these organoids and result in the development of a necrotic core. The two most common methods used to achieve vascularization in organoids are flow-based microfluidic systems and co-culture-based methods on co-permissive hydrogels.

Microfluidic systems induce organoid vascularization by providing a mechanical stimulus of blood flow (44). Designed ports and channels allow the delivery of nutrients and the removal of spent media, limiting waste accumulation. Complex microfluidic systems can include multiple cell types to carry out various functions. For example, ECs are perfused into the channels to create a cell lining that approximates a blood vessel. Vascular sprouting from the channels toward the organoid allows a microvascular bed to form within and around the organoid; this can be seeded onto or embedded within ECM (43). Alternatively, organoid vascularization can be induced by fluidic shear force (FSS) (44). In such models, microfluidic devices are equipped with two ports for fluid flow into and out of a central chamber. The bottom part of the chamber is coated with ECM gel and developing organoids are then allowed to attach prior to being subjected to high fluidic shear stress (FSS) (0.008–0.035 dyn/cm^2^). This system was originally designed for vascularizing kidney organoids derived from pluripotent stem cells where under flow, the endothelial progenitor cell pool expands to allow generation of vascular networks supported by mural cells (44). The authors saw that within 24 h, under high FSS, adjacent kidney organoids began to fuse, and novel vascular networks had formed to link them (44).

**Figure 3:**
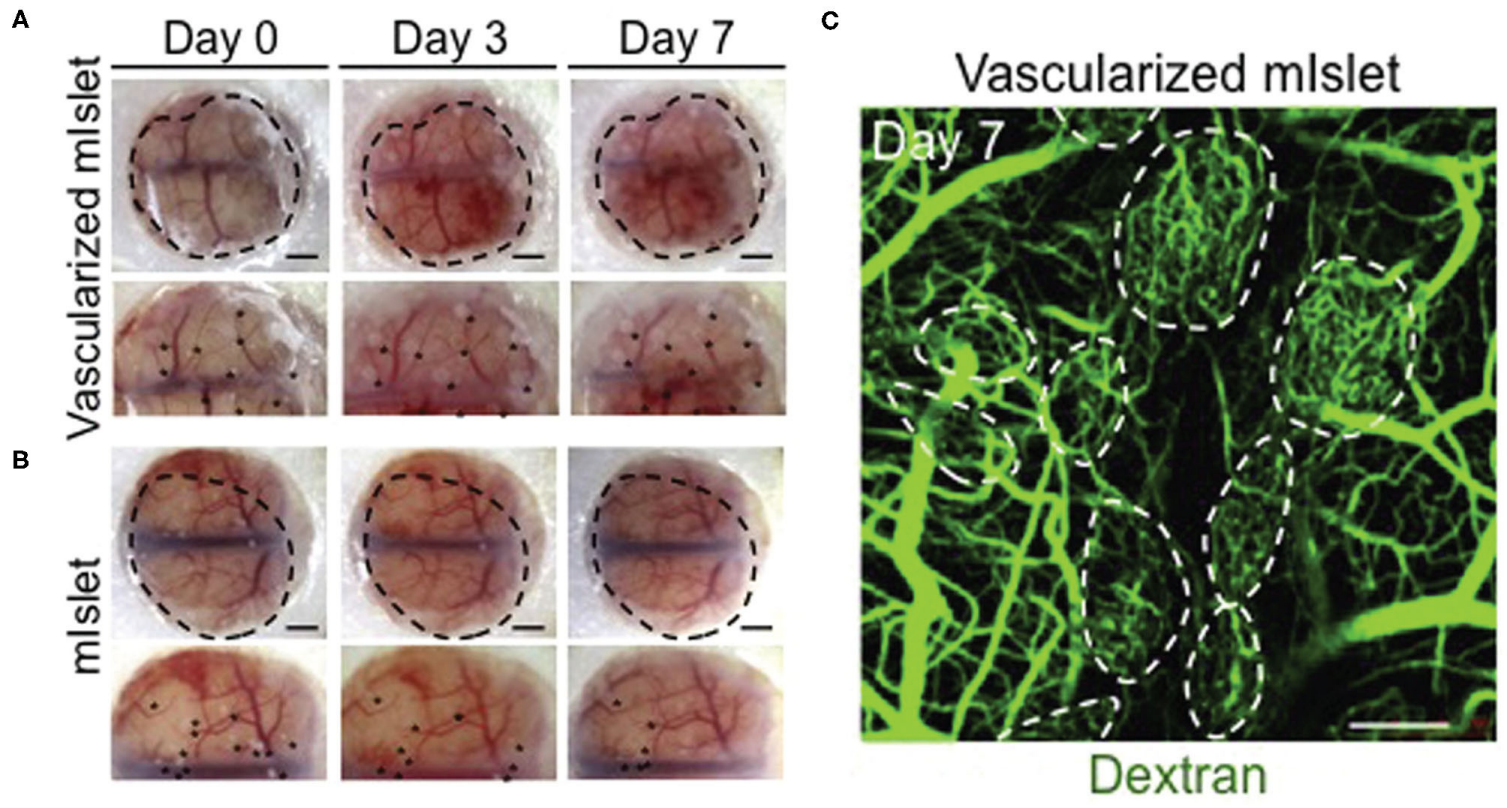
Isolated mouse islets were co-cultured with human MSCs and HUVECs and vascularization was achieved via MSC-dependent self-condensation *in vitro*. **(A)** Vascularized and **(B)** non-vascularized mouse islets were then transplanted under the renal capsule of diabetic mice. Black dots are indicative of the transplanted organoids. **(C)** Dextran was injected into tail veins at day 7 to visualize blood vessel lumens that formed within the organoids (dotted circles). Confocal stacks were obtained to visualize dextran-infused vessels. Adapted with permission from Takahashi et al. (46).

Organoid vascularization in co-culture relies on culturing stem cells with other cell types such as HUVECs, mesodermal progenitor cells (45), and mesenchymal stem cells (MSCs) (46). MSC-based self-condensation allows for MSCs and HUVECs to form aggregates that develop into vascularized organoids. In this context MSCs are used as feeder cells to provide sustained growth factor release and cell-cell contact for the developing organoid, as well as supporting cells for the developing microvasculature within the organoid. This is successful for liver and islet organoid systems (Figure 3), and 3D MSC/HUVEC spheroids *in vitro* (46–48). The resultant vascularized liver and islet organoids successfully inosculated *in vivo*. The MSC/HUVEC spheroids encapsulated in collagen/fibrin gel were used for the purpose of bone regeneration and demonstrated enhanced cell viability, osteogenic differentiation, and improved bone-mineral deposition.

A vascular network is not only limited to endothelial tubes. Larger blood vessels are comprised of multiple layers of smooth muscle cells lined by ECs. Capillary networks as well require support from mural cells known as pericytes to assist in contractility and maturation. More recently, it has been demonstrated that mesodermal progenitor cells (MPCs) have the potential to form hierarchical vascular networks by differentiating into multiple cell types such as ECs, smooth muscle cells, and mural cells (45, 49). Using MPCs for vascularized organoid and vascular network development can benefit from higher plasticity compared to the use of MSCs and HUVECs.

Self-assembled vasculature in vascularized organoids can bridge a gap in the field of tissue engineering by achieving vascular network parameters that are yet to be achieved using engineering techniques. Combining the biological benefits of vascularized organoids with advanced engineering technologies can potentially accelerate functional tissue growth and vascularization. For example, organoids can be used for bioprinting purposes to construct engineered tissue with a hierarchical vascular organization. In this process, sacrificially printed microchannels are directly embedded in a organoid-based hydrogel that resembles the granular tissue matrix. Microchannels are then perfused with ECs to form a tube-like structure resembling a large main vessel (39). Organoids are also capable of fusing with each other to form larger aggregates (44, 50). There is the potential that organoids, if adequately vascularized, can fuse to allow reconstruction of larger organs that can eventually replace defective ones.

## Using Elastin-Derived Materials in Fabricating Microvasculature

Interactions between cells and matrix proteins alters matrix stiffness and remodels the matrix. Hence, ECM-mimicking materials that are amenable to vascularization *in vitro* and *in vivo* must exhibit viscoelastic properties that enable them to dissipate stress in a relatively similar manner to that of native ECM (1). One way to reinforce these viscoelastic properties is through using elastin-derived peptides or tropoelastin, as these biopolymers offer various signaling and structural benefits. Since elastin is a critical component of an elastic ECM, elastin and elastin-based molecules that enhance the healing response, such as tropoelastin, can be incorporated into various engineering techniques to create microvasculature.

Elastin is recognized as a structural protein that helps to define the architecture and biomechanical properties of ECM but less appreciated is the fact that it also provides association and signaling benefits through interactions with elastin receptor complexes (ERC), and integrins (51). For example, tropoelastin and elastin-derived materials such as elastin-like polypeptides and elastin-like recombinamers (52–55) can promote early angiogenesis by providing integrin receptor interactions such as α_V_β_3_ (56) and α_V_β_5_ (57) for fibroblasts and ECs to attach, proliferate and migrate, resulting in angiogenesis (58–60). These angiogenic effects extend to MSC/EC co-culture systems in which tropoelastin is used a signaling molecule to promote stable vascular network formation (61). Tropoelastin can also enhance the biological behavior of MSCs during culture while maintaining stemness (62). Because of the cell-interaction benefits imparted by tropoelastin, incorporating it in MSC-dependent self-condensation methods has the potential to enhance MSC function and improve vascularization of organoids.

Elastin can be degraded by multiple matrix metalloproteinases (MMPs) such as MMP-2, 7, 9, 12 and 14, allowing for subsequent remodeling. For engineered materials, remodeling can be achieved through the use of the elastin-derived peptide VGVAPG. This peptide modulates ECM degradation through the upregulation of membrane-type metalloproteinase-1 (MT1-MMP) on the surface of ECs. MT1-MMP activates pro-MMP-12 to degrade the ECM and create spaces for nascent capillary tubes (56). This benefit can be harnessed by using elastin-derived materials as the main constituent such as in porous scaffold or in a hydrogel system for bioprinting. For example, by combining either soluble or insoluble elastin fibers with a collagen porous scaffold, the number, size and density of blood vessels formed within the scaffold is superior to the those without elastic fibers (63). As for bioprinting systems, tropoelastin-based hydrogel, a MeTro/GelMA system with shear-thinning properties, is useful for extrusion bioprinting. The biocompatible hydrogel can be crosslinked following exposure to a UV or visible light source, allowing the resulting construct to house multiple cell types (64).

The properties of the materials that are used to fabricate vascularized tissue constructs should tolerate traction forces exerted by cells to support remodeling. The presence of vasculature in any system requires some degree of elasticity and compliance for functioning. Although capillaries themselves do not rely on elastin, arterioles that give rise to capillaries do. For achieving hierarchical vessel organization, it might be beneficial to provide cells with matrix components that contribute to their biological function. Elastin-derived materials should be further explored as a material for engineering microvasculature.

## Conclusions and Future Perspectives

Scaffolds are generally designed from materials that can support cell attachment and are amenable to remodeling to facilitate regeneration and vascularization. Typically, cells that attach onto the scaffold release degradative enzymes to create space for nascent capillary tubes. Engineering microvascular channels into scaffolds for tissue engineering purposes offers spatiotemporal control by creating ports for ECs to facilitate angiogenesis, as a way of optimizing scaffold geometry. This maximizes the vascularization potential of the scaffold and minimizes the time required for angiogenic events to progress within the scaffold. However, endothelialization does not simply imply that the formed networks are functional and mature. Mature networks are mainly characterized by stable junctions, basement membrane deposition, mural cell support, and lumen formation (7, 37). Vessel integrity in engineered vascularized scaffolds is often assessed via injection of fluorophores to visualize flow behavior at newly vascularized sites. Rarely, other aspects examining vessel maturity are assessed.

Each of the techniques discussed above exhibits limitations that can be addressed by combining multiple approaches or by modifying existing approaches. Combining conventional techniques such as gas foaming and salt leaching to generate sponge-like scaffolds enhances the interconnection between pores over each technique on its own. Despite the benefits offered by combining these techniques, careful control over pore size and morphology is a persistent problem and the potential presence of porogen residuals compromises the potential biological applications of porous scaffolds. Melt-electrospinning has recently regained attention in the biomedical field for its promising applications. It provides further control over pore size by enabling precise fiber placement, as opposed to random fiber deposition in traditional electrospinning techniques, while benefiting from the lack of toxicity as no solvent is required for solubilization.

The appropriate choice of biomaterials presents the biggest challenge in 3D bioprinting. Ink properties must be compatible with cells, the 3D printer, and the final purpose of the tissue construct. Constraints such as temperature, pressure and cross-linking impose challenges in 3D bioprinting. In extrusion printing, shear forces can compromise cell viability and preclude their use with sensitive cell types. The incorporation of cells is also limited in stereolithography due to the extended UV-exposure times for polymerization.

Despite developing techniques to create channels for microvasculature formation, using polymers that impart biological benefits such as promoting attachment and enhancing angiogenic responses is crucial. Traditionally, growth factors and cytokines are incorporated into the scaffold to enhance cell infiltration and vascularization. However, the biological response is limited to the amount of growth factors present. It is therefore preferred that the various signaling molecules or polymers used in the engineered construct allow for directed or sustained release of growth factor and cytokines. Exploring cell responses to natural polymers such as elastin-based materials is important for informing on future biomaterial designs for engineered complex microvasculature.

## Author Contributions

AA, ZW, LL, and MZ research and wrote the manuscript. AW advised on and edited the manuscript. All authors contributed to the article and approved the submitted version.

## Conflict of Interest

AW is the founding scientist of Elastagen Pty. Ltd., now sold to Allergan, Inc., an Abbvie company. The remaining authors declare that the research was conducted in the absence of any commercial or financial relationships that could be construed as a potential conflict of interest.
